# Dementia-Preventing Behavior Awareness and Uptake Rates among Japanese Women in Midlife: A Survey-Based Pilot Study

**DOI:** 10.3390/ijerph191610029

**Published:** 2022-08-14

**Authors:** Yukiko Suzuki, Nanako Yamane, Kanto Tsukagoshi, Mina Yamaguchi, Hideki Mochizuki

**Affiliations:** 1 Department of Occupational Therapy, Kyorin University, Shimorenjyaku 5-4-1, Mitaka, Tokyo 181-8612, Japan; 2 Graduate School of Comprehensive Scientific Research, Prefectural University of Hiroshima, Ujinahigashi 1-1-71, Minami-ku, Hiroshima-shi 734-8558, Japan; 3 Akabane Rehabilitation Hospital, Akabanenishi 6-37-12, Kita-ku, Tokyo 115-0055, Japan; 4 Meirikai Chuo General Hospital, Higashijyujyo 3-2-11, Kita-ku, Tokyo 114-0001, Japan

**Keywords:** dementia, preventive behavior, midlife

## Abstract

Lifestyle changes may help prevent dementia. However, the perception and practice of dementia-preventing behaviors remain unclear; understanding both factors is required to help prevent dementia already at early stages. This study aimed to examine the awareness and uptake rates of dementia-preventive behaviors among community-dwelling women aged 40 to 64 years, and their associations with dementia-related anxiety. A self-administered anonymous questionnaire was distributed by mail from January to May 2020. The effective response rate was 20.4% (*n* = 47). Approximately 60% of the responders had dementia-related anxiety; approximately 80% wanted to prevent dementia. The participants were aware of two or more dementia-preventive behaviors; however, less than 50% of them practiced at least one behavior. The group with dementia-related anxiety was more interested in and aware of dementia prevention methods than the group without the anxiety. Women with greater dementia knowledge also knew more methods of preventing it; however, they were not necessarily implementing the recommended behaviors.

## 1. Introduction

The number of people with dementia is increasing globally [1], making it a public health concern. Genetic and age-related factors may increase the risk of dementia; lifestyle diseases (such as hypertension, dyslipidemia, and diabetes) may also increase this risk [2]. In fact, the increasing rates of lifestyle-related diseases have been linked to the growing incidence of dementia, in particular, in middle-aged populations [3,4]. Geda et al. [5] interviewed 1324 older adults about their exercise levels in mid- and in late-life, reporting that moderate exercise decreased the odds of mild cognitive impairment at both stages. Rovio et al. [6] conducted follow-up surveys of 1449 older adults on leisure physical activities in middle age, showing that performing at least two physical activities per week in middle age significantly reduced the odds of developing dementia. Moreover, Kivipelto et al. [7] showed that the risk of developing Alzheimer’s disease increased over six-fold in patients with obesity, hypertension, and hypercholesterolemia in midlife (aged 40 to 55 years), compared with that of their counterparts. This evidence suggests that lifestyle changes may help prevent dementia. However, the perception and practice of dementia-preventing behaviors remain unclear; an understanding of both factors is required to help prevent dementia at its early stages.

Dementia awareness is increasing with its incidence; in fact, older adults report high levels of interest in dementia [8], and many middle-aged individuals experience dementia-related anxiety [9]. Individuals with dementia-related anxiety tend to be aware of many dementia-preventive behaviors and may practice some of them. Midlife is a period of many physical, social, familial, and psychological changes and may include existential questioning and crises. Mental distress in middle age may increase the risk of developing dementia later [10,11,12]. It may also be associated with physical and mental health status at an older age. Understanding factors associated with dementia-related anxiety in midlife may inform intervention development.

Previous studies have reported differences in anxiety among midlife individuals according to sex [13,14,15]. Women report more intense, more numerous, and more frequent bodily symptoms than men [14]. Brody et al. [15] reported that women were almost twice as likely as men to have depression. Therefore, to avoid sex bias, this study included female individuals only.

There have been few studies on the perception and practice of dementia-preventing behaviors from the viewpoint of lifestyle [16,17,18]. A systematic review conducted by Cations et al. in 2018 [18] identified 34 studies on the knowledge of preventive and treatment measures for dementia, finding that there was insufficient knowledge of both topics. Of the 34 studies, 27 investigated knowledge of dementia-prevention; however, they all targeted a wide range of the general population, and none focused solely on midlife women. Yeo et al. [16] reported no link between concerns about dementia and social class, sex, intelligence scores, and age. However, no studies have investigated the relationship between concern about dementia and dementia-preventive behaviors.

This study surveyed Japanese women in midlife, aiming to understand the awareness and uptake rates of dementia-preventing behaviors. Japan is among the fastest aging societies worldwide. Herein, we evaluated dementia-related anxiety levels, interest in preventing dementia, and awareness and uptake rates of dementia-preventing behaviors. This study also evaluated the relationship between dementia-related anxiety and awareness, and the uptake levels of dementia-preventing behaviors.

## 2. Materials and Methods

### 2.1. Operationally Defined Terms

In this study, individuals in “midlife” were defined as community-dwelling individuals aged 40 to 64 years. Lazarus [19] defined anxiety as an emotion that arises from the perception of a situation as surprising and relevant to oneself. Dementia anxiety was defined as an “unpleasant emotion that may arise while considering the possibility of suffering from dementia in the future”. “Dementia-preventing behaviors” were defined as an “understanding of dementia risk at early stages and engaging in behaviors that may prevent or delay dementia onset” [17].

### 2.2. Study Design and Participants

When the sample size was calculated using G-Power, with an effect size of 0.8, a *p*-value less than 0.05, and a power of 0.8, the Mann–Whitney U-test required 27 participants per group. The sample size for this study was close to this target because the analysis was conducted based on data from 47 participants.

To efficiently collect participants, this study employed a relationship recruiting method. Parents who were willing to participate in the survey were recruited from four classes of college students at the university. The survey instructions and questionnaires were mailed to the parents of medical college students; the parents were assumed to be in the middle-aged group. Among those who responded to the survey, 47 women aged 40 to 64 years who were residents of the community were selected and included in the analysis.

### 2.3. Evaluation

The questionnaire interrogated participant characteristics, dementia-related anxiety levels, concerns over dementia prevention, dementia knowledge, and dementia prevention awareness and practice.

Participant characteristics included sex, age, household composition, years of education after primary school graduation, number of diseases for which the participant was currently receiving treatment, number of oral medication types prescribed, caregiving experience for individuals with dementia, and contact with relatives who are experiencing dementia.

To evaluate dementia anxiety, we asked participants to respond to the statement, “I am anxious about developing dementia in the future”, using responses such as, “I do not feel anxious at all”, “I do not feel anxious”, “I am not sure”, “I rarely feel anxious”, “I sometimes feel anxious”, or “I always feel anxious”. Responses including “rarely”, “sometimes”, and “always” were categorized as “dementia anxiety”; the remaining responses were categorized as “no dementia anxiety”. The sample was dichotomized into anxiety and non-anxiety groups.

Concerns about dementia prevention were evaluated with the following question: “I am concerned about dementia prevention”. The corresponding responses were: “I am very concerned”, “I am somewhat concerned”, “I am not sure”, “I am not very concerned”, and “I am not concerned at all”. The corresponding scores were 5, 4, 3, 2, and 1 point(s), respectively.

Dementia knowledge was assessed using the Attitude toward Dementia and Dementia Knowledge Scale [20]. This scale’s items include general knowledge about dementia, behavioral and psychological symptoms of dementia, and how to cope with the symptoms. Its validity and reliability for determining an individual’s knowledge of dementia has been confirmed. In addition, since it is a self-administered questionnaire, it can be used for mail surveys. This scale evaluates general knowledge regarding dementia and responses to the behavioral and mental symptoms of dementia by using the following statements: “I think so” (1 point), “I do not think so” (0 points), and “I do not know” (0 points). The total score ranges from 0 to 15 points; the higher the score, the greater the responder’s knowledge about dementia.

Awareness of dementia-preventing behaviors was evaluated with the question, “What behaviors do you think are effective at preventing the onset of dementia?” accompanied by a list of possible responses to select from (multiple choices allowed) or a free text field. The frequencies of reported behaviors were counted. We also asked participants about their information sources (multiple choices allowed); the frequencies of reported sources were counted.

To evaluate the participants’ practice of dementia-preventing behaviors, we asked an open-ended question: “What kind of behavior are you practicing to prevent the onset of dementia?” Participants who did not practice any dementia-preventing behaviors were asked to provide their reasons in a free-text form. With respect to the behaviors practiced, the number of different forms of actions was counted by collecting together similar information from the free-text form according to the classification of Tanaka et al. [17]. The number of behaviors practiced was compared between the dementia-related anxiety and no-anxiety groups.

### 2.4. Data Analysis

For concerns regarding dementia anxiety, we calculated the rates of dementia anxiety. The rates of awareness and uptake of dementia-preventing behaviors were compared between the groups with and without dementia-related anxiety. The participants’ characteristics were compared between the groups. The Shapiro–Wilk test was used to confirm the normality of distribution of variables, such as age and the number of dementia-preventing behaviors practiced. Other variables did not follow a normal distribution. Normally distributed variables were compared using the *t*-test. Non-normally distributed variables were compared with the χ^2^ test, Fisher’s exact test, or Mann–Whitney U-test. 

In addition, Spearman’s rank correlation coefficients were used to evaluate relationships among dementia awareness, prevention, and practice rates, sources of information, the amount of knowledge about dementia, and the types of behaviors practiced. SPSS (Ver. 21.0, IBM Corporation, Armonk, NY, USA) was used for statistical analyses, and the significance level was set as a *p*-value of < 0.05.

### 2.5. Ethics Statement

This study was approved by the institutional review board of the Faculty of Health Sciences, Kyorin University (approval number: 2019-52). The mailed surveys were accompanied by a study information sheet; this explained the purpose of the investigation and its voluntary nature, providing details of how data would be anonymized, managed, and stored. Data collection was performed when the respondent returned the completed questionnaire and consent form to the study director. This study was conducted in accordance with ethical guidelines.

## 3. Results

### 3.1. Questionnaire Recovery

In this study, women who were between 40 and 64 years of age (middle-aged) were selected among the respondents. We distributed a total of 230 questionnaire copies to local residents (parents of students enrolled in a medical university in Japan) by mail; 90 were correctly filled out and returned. Seven respondents with missing data and eight participants who were older than 65 years, as well as 28 male respondents, were excluded from the analysis; thus, data from 47 participants (effective recovery rate: 20.4%) were included in the analysis. The survey was conducted from January 2020 to May 2020.

### 3.2. Participant Characteristics

Participant characteristics and evaluation results are shown in Table 1. The survey included a total of 47 participants (mean age, 51.4 ± 4.3 years), and the mean number of years of education after primary school graduation for all the participants was 8.8 ± 1.4 years. Twelve (25.5%) participants had medical conditions, and one (2.1%) had orthopedic diseases (multiple answers). Eleven (23.4%) participants had experience in caring for people with dementia, and 20 (42.6%) had contact with a relative with dementia. 

Twenty-nine participants (mean age, 51.5 ± 4.4 years) reported having dementia-related anxiety, while 18 (mean age, 51.2 ± 4.3 years) reported no dementia-related anxiety. Thirty-eight (80.9%) participants were “very” and “somewhat” concerned with dementia prevention.

The groups with and without dementia anxiety had similar characteristics.

### 3.3. Recognition of Dementia-Preventive Behaviors and Their Practice

All the participants were aware of two or more dementia-preventing behaviors. The specific dementia-preventing behaviors identified by the participants in descending order of frequency were as follows: trying to interact with people/social participation (conversation and joining a social group: *n* = 47, 100%), self-directed awareness and living a developed lifestyle (having a purpose in life and trying new things: *n* = 47, 100%), moving the body (jogging and strength training: *n* = 46, 97.9%), diet and health management (paying attention to nutritional balance and sodium reduction: *n* = 46, 97.9%), brain-based games (such as puzzles and game of go: *n* = 46, 97.9%), writing (keeping a diary and drawing: *n* = 45, 95.7%), engaging in hobbies (playing an instrument and watching a movie: *n* = 45, 95.7%), reading (books and newspapers: *n* = 45, 95.7%), engaging in manual tasks (braiding and playing the piano: *n* = 44, 93.6%), doing housework (cooking and laundry: *n* = 42, 89.4%), recalling events of the previous day (*n* = 36, 76.6%), and other activities (such as dressing up: *n* = 2, 4.3%) (Table 2).

Approximately 46.8% of the participants practiced one or more preventive behaviors. The practiced behaviors, in descending order of frequency, were moving the body (walking, stretching, and swimming: *n* = 12, 25.5%), trying to interact with people/social participation (work, volunteering, and conversation: *n* = 8, 17.0%), diet and health management (eating a healthy diet and getting enough sleep: *n* = 6, 12.8%), brain-based games (such as Sudoku and studying: *n* = 5, 10.6%), doing housework (cooking: *n* = 4, 8.5%), reading (books and newspapers: *n* = 3, 6.4%), engaging in manual activities (such as handicrafts and playing the piano: *n* = 3, 6.4%), and other activities (such as living with goals and challenging oneself to do new things: *n* = 5, 10.6%) (Table 2).

Meanwhile, 25 (53.2%) participants did not practice any preventive behavior. Their reasons for not practicing any preventive behaviors included “no sense of urgency”, “no sense of need to take action”, “no need to take action while living a normal life”, “no time”, “no specific plan”, and “no reason”. 

### 3.4. Differences between Groups with and without Dementia Anxiety

The participants in the dementia-related anxiety group (61.7%) were more interested in dementia prevention than those in the no-anxiety group were (38.3%) (*p* = 0.043). The group with anxiety engaged in more preventive behaviors than the group without anxiety did (*p* = 0.015). Neither group showed any bias toward any specific behavior (Table 2). In addition, the levels of dementia-related knowledge were comparable in both groups. The number and type of dementia-preventing behaviors practiced were comparable in both groups (Table 2). 

### 3.5. Dementia Knowledge and Prevention

Among all the participants (*n* = 47), the number of behaviors recognized as dementia-preventing was correlated with the amount of knowledge on dementia (*r* = 0.341, *p* = 0.019). The number of behaviors recognized as dementia-preventing was correlated with the number of known information sources (*r* = 0.342, *p* = 0.019). There was no correlation between the number of behaviors recognized and those practiced (*r* = 0.032 *p* = 0.830). 

## 4. Discussion

### 4.1. Questionnaire Recovery

Despite the response rate of 39.1%, the effective recovery rate was low at 20.4%. Generally, it is recommended to evaluate the validity of the response rate by setting a guideline of 70% or higher [21]. However, Rubenfeld [21] suggested that a 70% response rate may not be attainable for some topics, and that a reasonable response rate may vary depending on the target population and the nature of the question. The fact that the topics in this study were focused on illness and personal matters may have discouraged respondents, resulting in a low response rate. Furthermore, the reason for the low effective response rate in this study was the high survey response rate among fathers of college students.

### 4.2. Dementia Anxiety and Prevention in Women in Midlife

The average age of the participants of this survey was 50 years. Approximately 60% of the participants had anxiety about dementia. Saito et al. [9] reported that approximately 80% of people in midlife have anxiety about getting dementia; the present study findings are consistent with those of this previous study. In this study, 80% of the participants were interested in dementia prevention; this suggests high levels of interest in dementia prevention in the study population.

### 4.3. Dementia Prevention Awareness and Practice

All the participants listed at least two types of behaviors that they considered effective at preventing dementia. The participants recognized some behaviors as effective at preventing dementia. Specific preventive actions included interacting with people, social participation, self-directed awareness and living a developed lifestyle, moving the body, and diet and health management. Fratiglioni et al. [22] suggested that a poor social network increases the risk of developing dementia, and dementia has been associated with social factors. In this survey, social participation was known to the participants as a dementia-preventing behavior. All the participants also perceived as a preventive action trying to live a positive life by themselves rather than relying on others. Psychological factors, such as depression, have been associated with the development of dementia [23,24]. Boyle et al. [25] showed a correlation between physical activity and dementia incidence in a longitudinal study of 1121 older adults. Overall, having a positive attitude may increase physical activity levels and help prevent dementia. In addition, approximately 90% of the participants perceived health management through exercise and diet as a dementia-preventing behavior; these perceptions are supported by evidence on physical exercise [5,6,26,27] and healthy diets [28].

Approximately 50% of the participants reported having implemented at least one dementia-preventing behavior; the corresponding rate in a previous study was 20% (age, 45.9 to 59.9 years) [29]. Since the participants of this study were parents of medical university students, their medical interests, knowledge, and motivations may be greater than those of the general population; this translates into relatively higher rates of dementia preventive action.

Approximately 30% of the participants reported engaging in physical activity, including walking and gymnastics, to help prevent dementia. In addition, approximately 20% of the participants had active social lives. Regional differences have been previously observed in interpersonal relationships and social participation; specifically, in cities with high population densities, the number of neighbors and relatives who interact with each other is small and the frequency of community participation is low [30,31]. Herein, most participants were residents of relatively densely populated cities. Thus, the 20% rate of social participation is not low.

Nevertheless, over 50% of the participants did not practice any dementia-preventing behavior; they quoted reasons such as “no urgency” associated with dementia onset and “no need” to prevent dementia. These findings suggest that dementia prevention awareness should increase to help affect lifestyle choices that may help prevent dementia [5,6,7].

### 4.4. Differences between Groups with and without Dementia Anxiety

The participants reporting dementia-related anxiety were more likely to be aware of and to engage in dementia prevention than their counterparts. In fact, high levels of dementia-related anxiety were associated with increased interest in and knowledge of dementia-preventing behaviors. The rates of engagement in dementia-preventing behaviors were comparable between the groups. Dementia-related information was easily accessible to the study participants both locally and online. Engagement in dementia-preventing behaviors may be determined by dementia perception rather than anxiety.

Dementia-related anxiety did not affect the type of dementia-preventing behaviors practiced. Borkovec [32] reported that worried people find positive meaning in worrying and tend to view it as a useful means of problem-solving. However, in this survey, there was no association between dementia anxiety and the practice of dementia-preventive actions to solve this anxiety. Women in midlife tend to be busy with both child-rearing and professional activities; in addition, even if they have dementia anxiety, their ability to practice dementia-preventing behaviors may be restricted by circumstances. Therefore, anxiety about dementia may not always trigger the motivation to practice preventive behaviors. Future studies should examine environmental and social factors that prevent women in midlife from engaging in dementia-preventing activities.

### 4.5. Dementia Knowledge and Prevention

The number of dementia-preventing behaviors practiced and recognized and the levels of dementia-related knowledge were correlated. The participants who reported engaging in a high number of dementia-preventing behaviors tended to report having access to a large number of relevant information sources. However, the number of preventive actions recognized was not associated with that of actions practiced; this suggests that knowledge may not be enough to act. Consequently, while access to information is increasing, it is necessary to create environments that support dementia prevention, providing time, space, and social support that encourage engagement.

## 5. Strengths and Limitations

Our study had some limitations. First, the sample size was small, which may limit the generalizability of our findings. Further studies with larger samples are required to draw more generalizable conclusions. Second, the participants were the parents of medical university students; their dementia awareness levels may have been higher than those of the general population. Selection bias may have affected the presented findings, as the sample included women interested in and knowledgeable about medicine and dementia prevention. Future studies should include women with and without children sampled from the general population. Third, the participants had the option to report dementia-preventing behaviors they engaged in as a free text; consequently, some of the practices may have lacked an evidence base. The present study findings should be interpreted with caution. Future studies should use standardized objective measures to assess the awareness and uptake of dementia-preventing behaviors.

## 6. Conclusions

More than half of the study participants reported dementia-related anxiety. Women in midlife who report having dementia-related anxiety have a high interest in dementia prevention and are aware of dementia-preventing behaviors. Nevertheless, fewer than half of the participants engaged in dementia-preventing behaviors. Further studies are required to elucidate barriers to engaging in dementia-preventing behaviors despite dementia awareness. Social and environmental factors affecting the uptake of dementia-preventing behaviors should be examined.

## Figures and Tables

**Table 1:** Characteristics of the participants.

		Total (*n* = 47)	Dementia Anxiety Group (*n* = 29)	No Dementia Anxiety Group (*n* = 18)	*p*-Value
Age, years		51.4 ± 4.3	51.5 ± 4.4	51.2 ± 4.3	0.823
Household composition	Households living with children	30 (63.8%)	22 (75.9%)	8 (44.4%)	
	Spouse-only households	11 (23.4%)	5 (17.2%)	6 (33.3%)	
	Households living with parents	2 (4.3%)	0 (0%)	2 (11.1%)	
	Other	4 (8.5%)	2 (6.9%)	2 (11.1%)	
Education, years		9 (6, 11)	10 (6, 11)	8 (6, 10)	0.069
Comorbidities, *n*		0 (0, 2)	0 (0, 2)	0 (0, 2)	0.818
Types of oral medicines, *n*		0 (0, 3)	0 (0, 3)	0 (0, 3)	0.951
Caregiving experiences of person with dementia †	Present	11 (23.4%)	8 (17.0%)	3 (6.4%)	0.492
Relatives with dementia †	Present	20 (42.6%)	13 (27.7%)	7 (14.9%)	0.767
Concern about dementia prevention		4 (2,5)	4 (2,5)	4 (2, 5)	0.043 *
Dementia knowledge score ^a^		9 (2, 12)	9 (5, 11)	9 (2, 11)	0.991
Recognition of dementia prevention, *n*		33.4 ± 12.8	36.9 ± 11.2	27.8 ± 13.5	0.015 *
Number of information sources		3 (0, 8)	3 (0, 6)	2 (0, 8)	0.198
Behaviors against dementia prevention, *n*		0 (0, 7)	1 (0, 7)	0 (0, 3)	0.174

†: Fisher’s exact test, other items: Mann–Whitney U test, ^a^ range: 0–15 points; the higher the score, the better the knowledge of the participants. The values are presented as means ± SDs or medians (ranges), unless otherwise stated. * *p* < 0.05, age, recognition of dementia prevention compared using the two-sample *t*-test (mean value ± standard deviation).

**Table 2:** Behaviors recognized as dementia-preventing and preventive behaviors practiced.

		Total (*n* = 47)	Dementia Anxiety Group (*n* = 29)	No Dementia Anxiety Group (*n* = 18)	*p*-Value
Recognized preventive behaviors (multiple answers)	Trying to interact with people/social participation	47 (100%)	29 (100%)	18 (100%)	-
	Self-directed awareness and living a developed lifestyle	47 (100%)	29 (100%)	18 (100%)	-
	Moving the body	46 (97.9%)	28 (96.6%)	18 (100%)	1.000
	Diet and health management	46 (97.9%)	28 (96.6%)	18 (100%)	1.000
	Brain-based games	46 (97.9%)	29 (100%)	17 (94.4%)	0.383
	Writing	45 (95.7%)	27 (93.1%)	18 (100%)	0.517
	Hobbies	45 (95.7%)	29 (100%)	16 (88.9%)	0.142
	Reading	45 (95.7%)	28 (96.6%)	17 (94.4%)	1.000
	Manual activities	44 (93.6%)	28 (96.6%)	16 (88.9%)	0.549
	Doing housework	42 (89.4%)	27 (93.1%)	15 (83.3%)	0.357
	Recall	36 (76.6%)	21 (72.4%)	15 (83.3%)	0.492
	Other	2 (4.3%)	1 (3.4%)	1 (5.6%)	1.000
Preventive actions in practice (multiple answers)	Moving the body	12 (25.5%)	8 (27.6%)	4 (22.2%)	0.744
	Trying to interact with people/social participation	8 (17.0%)	7 (24.1%)	1 (5.6%)	0.103
	Diet and health management	6 (12.8%)	5 (17.2%)	1 (5.6%)	0.384
	Brain-based games	5 (10.6%)	5 (17.2%)	0 (0%)	0.141
	Doing housework	4 (8.5%)	4 (13.8%)	0 (0%)	0.283
	Reading	3 (6.4%)	1 (3.4%)	2 (11.1%)	0.549
	Manual activities	3 (6.4%)	3 (10.3%)	0 (0%)	0.276
	Other	5 (10.6%)	5 (17.2%)	0 (0%)	0.141

The values are presented as counts (%). Comparisons were performed using the χ^2^ test or Fisher’s exact test.

## Data Availability

Data that support the findings of this study are available on reasonable request from the corresponding author.
